# Dysregulation of lysosomal morphology by pathogenic LRRK2 is corrected by TPC2 inhibition

**DOI:** 10.1242/jcs.164152

**Published:** 2015-01-15

**Authors:** Leanne N. Hockey, Bethan S. Kilpatrick, Emily R. Eden, Yaping Lin-Moshier, G. Cristina Brailoiu, Eugen Brailoiu, Clare E. Futter, Anthony H. Schapira, Jonathan S. Marchant, Sandip Patel

**Affiliations:** 1 Department of Cell and Developmental Biology, University College London, Gower Street, London, WC1E 6BT, UK; 2 Department of Cell Biology, Institute of Ophthalmology, University College London, London, EC1V 9EL, UK; 3 Department of Pharmacology, University of Minnesota Medical School, Minneapolis, Minnesota, 55455, USA; 4 Department of Pharmaceutical Sciences, Thomas Jefferson University, Jefferson School of Pharmacy, Philadelphia, 19107, USA; 5 Department of Pharmacology and Center for Substance Abuse Research, Temple University School of Medicine, Philadelphia, 19140, USA; 6 Department of Clinical Neurosciences, Institute of Neurology, University College London, London, NW3 2PF, UK

**Keywords:** Ca^2+^, LRRK2, Lysosomes, NAADP, Parkinson disease, TPCN2

## Abstract

Two-pore channels (TPCs) are endolysosomal ion channels implicated in Ca^2+^ signalling from acidic organelles. The relevance of these ubiquitous proteins for human disease, however, is unclear. Here, we report that lysosomes are enlarged and aggregated in fibroblasts from Parkinson disease patients with the common G2019S mutation in LRRK2. Defects were corrected by molecular silencing of TPC2, pharmacological inhibition of TPC regulators [Rab7, NAADP and PtdIns(3,5)*P*_2_] and buffering local Ca^2+^ increases. NAADP-evoked Ca^2+^ signals were exaggerated in diseased cells. TPC2 is thus a potential drug target within a pathogenic LRRK2 cascade that disrupts Ca^2+^-dependent trafficking in Parkinson disease.

## INTRODUCTION

Two-pore channels (TPCs) are ubiquitous endolysosomal ion channels that mediate Ca^2+^ signals in response to the Ca^2+^ mobilising messenger nicotinic acid adenine dinucleotide phosphate (NAADP) (Brailoiu et al., 2009; Calcraft et al., 2009; Hooper and Patel, 2012). The human isoforms, TPC1 and TPC2, target to discrete populations of acidic vesicles that comprise the endolysosomal system (Brailoiu et al., 2009; Brailoiu et al., 2010; Calcraft et al., 2009). These highly dynamic organelles undergo continual homo- and hetero-typic fusion in a Ca^2+^-dependent manner (Luzio et al., 2007). Fusion of lysosomes with endosomes or autophagosomes is crucial for endocytosis and autophagy. Proper functioning of lysosomes is also dictated by their number (Sardiello et al., 2009) and position (Korolchuk et al., 2011) within the cell. Lysosomal morphology might therefore serve as a sensitive read-out of endocytic well-being. Because TPCs regulate trafficking events within the endolysosomal system (Grimm et al., 2014; Lin-Moshier et al., 2014; Ruas et al., 2010; Ruas et al., 2014) there is the possibility that aberrant TPC activity could underlie endocytic dysfunction.

Parkinson disease is a progressive neurodegenerative disorder involving a complex aetiopathogenesis that includes several genetic causes and risk factors (Hardy, 2010; Schapira and Jenner, 2011). Mutations in *LRRK2* (also known as *PARK8*) are a cause of autosomal dominant familial Parkinson disease that is indistinguishable from sporadic forms (Healy et al., 2008; Paisán-Ruíz et al., 2004; Zimprich et al., 2004). LRRK2 is a large modular protein comprising both enzymatic domains (a ROC and kinase domain) and domains involved in protein–protein interactions (Cookson, 2010). The function of LRRK2 is not clear, but LRRK2 localises, at least in part, to the endolysosomal system (Alegre-Abarrategui et al., 2009; Biskup et al., 2006), and a number of studies (albeit using recombinant systems and animal models) implicate LRRK2 in endolysosomal trafficking and associated processes such as endocytosis and autophagy (Dodson et al., 2012; Gómez-Suaga et al., 2012; MacLeod et al., 2013; Shin et al., 2008).

Here, we examined endolysosomal morphology in fibroblasts from Parkinson disease patients with the common LRRK2 G2019S mutation. We identify pronounced lysosomal morphology defects, which were reversed by inhibition of TPC2 and associated regulators. Our data thus suggest that TPC2 acts downstream of pathogenic LRRK2 to regulate trafficking within the endolysosomal system in a pathway of potential relevance to the pathology of LRRK2-mediated Parkinson disease.

## RESULTS AND DISCUSSION

### Lysosomal morphology is disrupted in LRRK2 G2019S patient fibroblasts

**Fig. 1.**
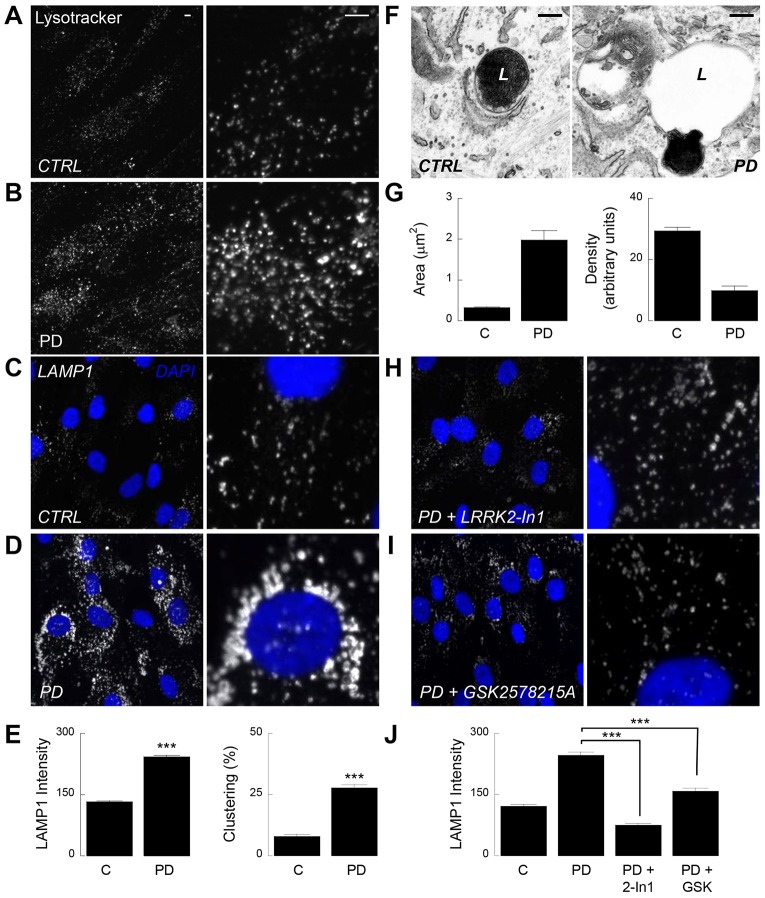
**Pathogenic LRRK2 disrupts lysosomal morphology in a kinase-dependent manner.** (A,B) Confocal images of Lysotracker® red fluorescence in live fibroblasts derived from a healthy control (A) and a Parkinson disease (PD) patient harbouring the LRRK2 G2019S mutation (B). Higher magnification images are shown in the right panels. Scale bars: 5 µm (and also apply to B–D,H,I). Fluorescence intensity was increased 1.4±0.07-fold (mean±s.e.m.) in Parkinson disease cells (*n* = 108 cells from three independent platings of two patient and paired control lines). (C,D) Confocal images of LAMP1 staining in fixed fibroblasts. Nuclei (stained with DAPI) are shown in blue. (E) Pooled data quantifying LAMP1 intensity (left) or the proportion of cells displaying perinuclear lysosome clustering (right). Data (mean±s.e.m.) are from 969 healthy control and 1181 LRRK2-PD cells from 21 independent platings of a single patient and paired control line. (F) Representative electron micrographs of endolysosomes from a healthy (left) and LRRK2-PD (right) fibroblast. L, lysosome. Scale bars: 200 nm. (G) Pooled data quantifying lysosome area (left) and density (right). Data (mean±s.e.m.) are from 100 lysosomes. (H,I) LAMP1 staining in LRRK2-PD fibroblasts treated for three days with the LRRK2 kinase inhibitors LRRK2-In1 (100 nM, H) or GSK2578215A (32 nM, I). (J) Pooled data quantifying LAMP1 intensity for the cells shown in H and I (mean±s.e.m., *n* = 136–335 cells from five independent platings of two patient and paired control lines). ***P<0.001.

We examined the morphology of lysosomes in primary cultured fibroblasts from LRRK2 G2019S Parkinson disease patients (LRRK2-PD cells) using three independent methods. In the first analyses, we stained live cells with the fluorescent acidotrope, Lysotracker®. This probe can be used to infer lysosome volume, which has recently been validated as a novel biomarker for lysosomal storage disorders (te Vruchte et al., 2014). In healthy control fibroblasts, lysosomes were well resolved as puncta (Fig. 1A). In contrast, lysosomes appeared enlarged and clustered in age-matched LRRK2-PD fibroblasts (Fig. 1B).

In a second approach, we assessed lysosomal morphology in fixed cells by immunocytochemistry using a primary antibody raised to the late endosome and lysosome marker LAMP1. Again, lysosomes were enlarged in the patient fibroblasts (Fig. 1D) compared to controls (Fig. 1C). This defect was manifest as an approximate doubling in intensity of LAMP1 labelling in LRRK2-PD cells (Fig. 1E). We also noted a propensity of lysosomes to cluster close to the nucleus in the patient fibroblasts (Fig. 1D,E). Similar defects were obtained using cultures derived from three other patients when compared to healthy controls (supplementary material Fig. S1) although western blot analysis did not reveal any consistent change in total LAMP1 levels (supplementary material Fig. S2A).

In the final approach, we performed electron microscopy to resolve the morphology of individual lysosomes. An example of a lysosome from a healthy control fibroblast displaying the typical electron dense interior is shown in Fig. 1F. Lysosomes in fibroblasts from Parkinson disease patients were more heterogeneous, and were often swollen and characterised by large translucent areas (Fig. 1F). Quantification of lysosomes in random sections showed that the average area was increased approximately sixfold whereas density was decreased approximately threefold (Fig. 1G).

The G2019S mutation in LRRK2 falls within its kinase domain and is associated with increased kinase activity (West et al., 2005). We therefore examined the effect of LRRK2-In1, a recently described potent LRRK2 kinase inhibitor (Deng et al., 2011). As shown in Fig. 1H, lysosomal morphology in LRRK2-PD fibroblasts reverted to a normal appearance following a 3-day treatment with LRRK2-In1 (100 nM). Similar results were obtained upon shorter treatments (supplementary material Fig. S3). We also tested a structurally distinct LRRK2 kinase inhibitor, GSK2578215A (Reith et al., 2012). GSK2578215A (32 nM) also normalised lysosomal morphology in LRRK2-PD cells (Fig. 1I). Pooled data are presented in Fig. 1J. Taken together, we identified pronounced changes in lysosomal morphology in fibroblasts from LRRK2-associated Parkinson disease patients that are dependent on LRRK2 kinase activity.

### Lysosomal defects are reversed by silencing TPC2 but not TPC1

**Fig. 2.**
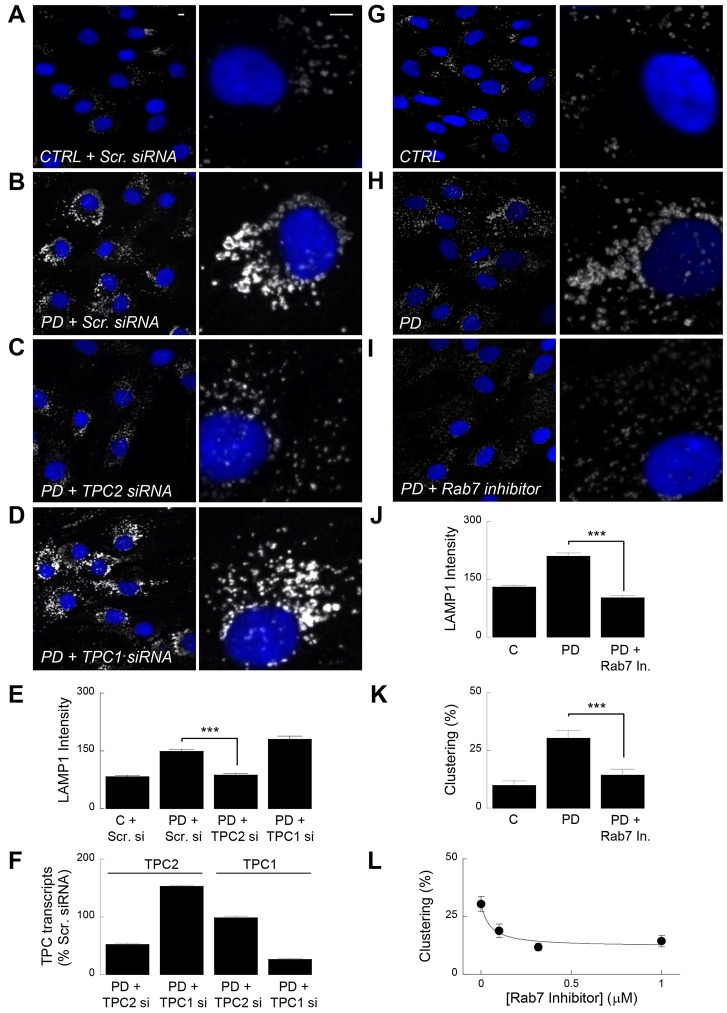
**TPC2 but not TPC1 mediates lysosomal morphology disturbances.** (A–D) LAMP1 staining in fibroblasts from a healthy control (CTRL) (A) and a Parkinson disease patient (PD) (B–D) treated with either a control siRNA (Scr. siRNA) (A,B) or siRNA to TPC2 (C) or TPC1 (D). Scale bars: 5 µm. (E) Pooled data quantifying LAMP1 intensity for the cells shown in A–D (mean±s.e.m., *n* = 381–532 cells from six independent knockdowns from two patient and paired control lines). (F) Quantitative PCR analysis of TPC2 (left) and TPC1 (right) levels in cells treated with the indicated TPC siRNA. Data are from two patients and are normalised to TPC levels in cells treated with scrambled control siRNA. (G–I) Lysosomal morphology in control fibroblasts (G) or LRRK2-PD fibroblasts treated without (H) or with (I) the Rab7 GTPase inhibitor CID 1067700 (1 µM, 3 days). (J,K) Pooled data quantifying LAMP1 intensity (J) or the proportion of cells displaying perinuclear lysosome clustering (K) for the cells shown in G–I (mean±s.e.m., *n* = 237–281 cells from five independent platings of three patient and paired control lines). ***P<0.001. (L) Concentration–effect relationship (mean±s.e.m., *n* = 130–281 cells from five independent platings of three patient lines) for the Rab7 GTPase inhibitor on lysosome clustering in LRRK2-PD fibroblasts.

The observed changes in lysosomal morphology in LRRK2-PD fibroblasts described here are reminiscent of those recently described upon overexpression of TPC2 (Lin-Moshier et al., 2014). To probe the role of TPCs in LRRK2 action, we used small interfering RNAs (siRNAs) to silence TPC expression in LRRK2-PD fibroblasts. As shown in Fig. 2A–C, lysosomal morphology was normalised in LRRK2-PD fibroblasts transfected with a siRNA against TPC2. Quantitative PCR confirmed selective knockdown of TPC2 transcripts in siRNA-treated cells (Fig. 2F). Intriguingly, we noted little effect of TPC1 silencing on lysosomal morphology in LRRK2-PD fibroblasts (Fig. 2D,E) despite demonstrable knockdown at both the transcript (Fig. 2F) and protein (supplementary material Fig. S2B) level.

Exaggerated perinuclear clustering of lysosomes in LRRK2-PD cells (Fig. 1) is consistent with the actions of both TPC2 (Lin-Moshier et al., 2014) and Rab7 (Bucci et al., 2000; Hutagalung and Novick, 2011). We therefore tested the effects of inhibiting Rab7 GTPase activity (Agola et al., 2012) on lysosomal morphology and distribution in LRRK2-PD fibroblasts. Importantly, lysosomal defects in LRRK2-PD cells were corrected upon Rab7 inhibition (Fig. 2G–K; supplementary material Fig. S3). This reversal was concentration dependent (Fig. 2L). Levels of endogenous VPS35, a component of the retromer complex (MacLeod et al., 2013), were unchanged in our LRRK2-PD fibroblasts compared to controls (supplementary material Fig. S2A). Taken together, data presented here reveal a specific role for TPC2 and a newly identified interactor (Rab 7) in regulating lysosomal morphology in LRRK2-PD cells.

### Lysosomal morphology defects are dependent on NAADP and PtdIns(3,5)*P*_2_

**Fig. 3.**
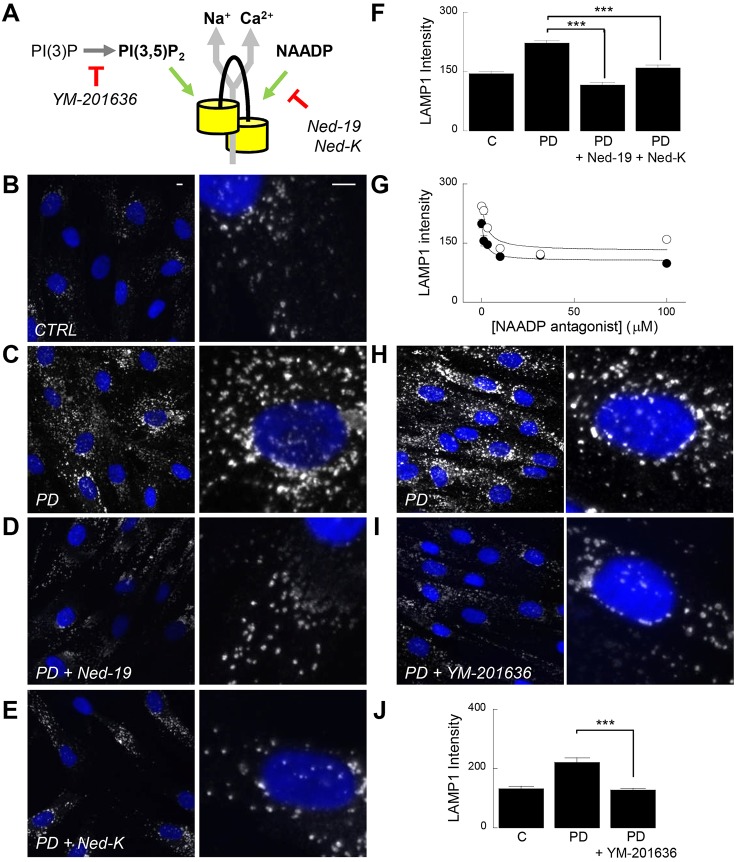
**Lysosomal defects are NAADP- and PtdIns(3,5)*P*_2_-dependent.** (A) Schematic of the TPC (yellow) showing proposed ion permeability (Ca^2+^ and Na^+^; grey arrows) and activating ligands (NAADP and PtdIns(3,5)*P*_2_; green arrows). Drugs used are highlighted in italics and their loci of action is shown in red. (B–E) LAMP1 staining in fibroblasts from a healthy control (CTRL) (B) and a Parkinson disease (PD) patient (C–E) treated for 3 days with either DMSO (B,C) or the NAADP antagonists, Ned-19 (100 µM, D) and Ned-K (100 µM, E). Scale bars: 5 µm. (F) Pooled data quantifying LAMP1 intensity for the cells shown in B–E (mean±s.e.m., *n* = 92–322 cells from six independent platings of three patient and paired controls). (G) Concentration–effect relationships (mean±s.e.m., *n* = 40–206 cells from six independent platings of three patient lines) for Ned-19 (black circles) and Ned-K (white circles) on lysosomal morphology in LRRK2-PD fibroblasts. (H,I) LAMP1 staining in LRRK2-PD fibroblasts treated without (H) or with the PIKfyve inhibitor YM-201636 (1 µM, 2 h) (I). (J) Pooled data quantifying LAMP1 intensity for the cells shown in H and I (mean±s.e.m., *n* = 73–292 cells from two independent platings of two patient and paired control lines). ***P<0.001.

Much evidence has accumulated identifying TPCs as the long-sought endolysosomal targets for NAADP (Brailoiu et al., 2009; Calcraft et al., 2009; Hooper and Patel, 2012; Zong et al., 2009). Notably, NAADP-induced Ca^2+^ release is inhibited when TPCs are silenced or genetically deleted (Brailoiu et al., 2009; Calcraft et al., 2009; Davis et al., 2012; Dionisio et al., 2011; Grimm et al., 2014; Lu et al., 2013). However, this view has been challenged by evidence suggesting that TPCs are not NAADP-sensitive channels but are instead Na^+^ channels gated by phosphatidylinositol 3,5-bisphosphate [PtdIns(3,5)*P*_2_] (Wang et al., 2012). We therefore examined the effects of antagonising the action of NAADP and PtdIns(3,5)*P*_2_ on lysosomal morphology in LRRK2-PD fibroblasts (Fig. 3A). Potential NAADP involvement was assessed using the NAADP antagonist Ned-19 (Naylor et al., 2009) and its novel analogue Ned-K (see Materials and Methods). As shown in Fig. 3B–F and supplementary material Fig. S3, lysosomal morphology in LRRK2-PD fibroblasts treated with either Ned-19 or Ned-K was similar to control cells. Reversal of defective lysosomal morphology by both compounds was dependent on concentration (Fig. 3G). Ned-19 and Ned-K thus mimicked the effects of TPC2 silencing (Fig. 2). We used the PIKfyve inhibitor, YM-201636 to deplete PtdIns(3,5)*P*_2_ levels (Jefferies et al., 2008). Acute treatment with the drug was also sufficient to reverse lysosomal morphology defects (Fig. 3H–J), again similar to the effects of silencing TPC2. Collectively, these molecular and pharmacological data suggest that TPCs are regulated by both NAADP and PtdIns(3,5)*P*_2_, findings consistent with recent reports (Grimm et al., 2014; Jha et al., 2014). These data also highlight the utility of mechanistically distinct small molecule inhibitors in correcting lysosomal pathology.

### Pathogenic LRRK2 disrupts local and global Ca^2+^ signalling

**Fig. 4.**
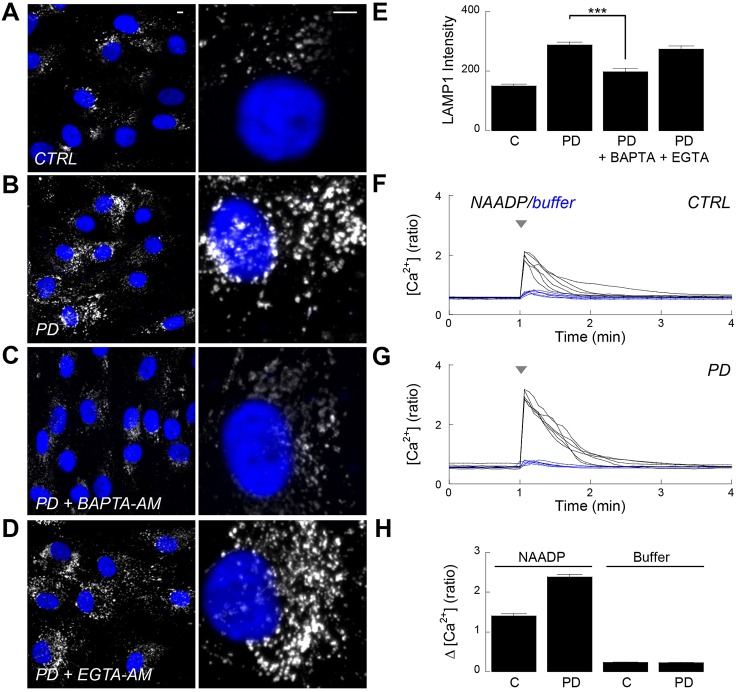
**Pathogenic LRRK2 disrupts local and global Ca^2+^ signalling.** (A–D) LAMP1 staining in fibroblasts from a healthy control (CTRL) (A) and a Parkinson disease patient (PD) (B–D) treated for 2 h with either DMSO (A,B) or 10 µM acetoxymethyl (AM) esters of the Ca^2+^ chelators BAPTA (C) or EGTA (D). Scale bars: 5 µm. (E) Pooled data quantifying LAMP1 intensity for the cells shown in A–D (mean±s.e.m., *n* = 150–307 cells from six independent platings of two patient and paired controls). ***P<0.001. (F,G) Cytosolic Ca^2+^ responses of individual Fura-2-loaded healthy (F) or Parkinson disease (G) fibroblasts microinjected with either vehicle (blue traces) or NAADP (20 µM pipette; black traces). (H) Pooled data (*n* = 6) quantifying the change in ratio upon microinjection of NAADP (left) or vehicle (right).

TPCs are ostensibly Ca^2+^-permeable, and constitutive Ca^2+^ release events within the endolysosomal system are known to regulate organelle fusion (Pryor et al., 2000). To probe the role of Ca^2+^ in lysosomal disturbances, we buffered Ca^2+^ levels using cell-permeable forms of either BAPTA or EGTA (Morgan et al., 2013). As shown in Fig. 4A–C and summarised in Fig. 4E, lysosomal morphology in LRRK2-PD fibroblasts acutely treated with BAPTA-AM were similar to control cells, suggesting that the morphological defects are dependent on Ca^2+^. However, treatment with EGTA-AM (a slower Ca^2+^ chelator) proved ineffectual (Fig. 4D,E). These data indicate that disrupted lysosomal morphology is likely due to dysregulated local Ca^2+^ signalling. This might promote Ca^2+^-dependent fusion of lysosomes (Pryor et al., 2000) and thus enlargement. Indeed, we often encountered large hourglass-shaped organelles delineated by a continuous membrane consistent with a fusion defect in Parkinson disease fibroblasts (supplementary material Fig. S4).

To further probe the role of Ca^2+^ in pathogenic LRRK2 action, we measured global cytosolic Ca^2+^ levels in response to NAADP stimulation. As shown in Fig. 4F, microinjection of fibroblasts with NAADP, but not vehicle, evoked Ca^2+^ signals. NAADP responses were significantly larger in LRRK2-PD fibroblasts than in healthy controls (Fig. 4G,H). These signals are likely the global correlate of enhanced TPC activity that underlies the trafficking defect. Thus, both local (constitutive) and global (NAADP-regulated) Ca^2+^ signals are disrupted upon LRRK2 mutation.

In summary, we identify trafficking defects in LRRK2-PD fibroblasts that result from interplay between TPC2, its regulatory interactors and ligands, and the associated Ca^2+^ fluxes. TPC2 and LRRK2 co-immunoprecipitate (Gómez-Suaga et al., 2012) raising the possibility that TPC2 is phosphorylated by LRRK2. Given that similar morphological changes within the endo-lysosomal system have been observed during normal ageing and in other neurodegenerative conditions (Nixon et al., 2008), we suggest that aberrant TPC signalling might have broader relevance to declining cellular function.

## MATERIALS AND METHODS

### Cell culture

Fibroblast cultures from four Parkinson disease patients carrying the G2019S mutation in LRRK2 and four healthy donors (each pair age-matched within 2 years; age range 48–71) were established as described previously (Papkovskaia et al., 2012). The study was approved by the Hampstead Research Ethics Committee. All individuals provided written informed consent for the provision of samples. Cells were maintained in DMEM supplemented with 10% (v/v) fetal bovine serum, 100 units/ml penicillin and 100 µg/ml streptomycin (all from Invitrogen) at 37°C in a humidified atmosphere with 5% CO_2_. Cells were passaged by scraping and plated onto glass coverslips (for confocal microscopy and Ca^2+^ imaging), thermanox coverslips (for electron microscopy) or directly onto tissue culture plates or flasks (for western blotting) before experimentation. Cultures were used between passage 6–15 and at 4–7 days post plating. Control and LRRK2-PD cultures were analysed in parallel and differed by no more than two passages.

### Drug treatment

All drugs used in this study were dissolved in DMSO, diluted into culture medium and the medium sterile filtered prior to use. The LRRK2 kinase inhibitors LRRK2-In1 and GSK2578215A were from Merck and R&D Systems, respectively. The Rab7 GTPase inhibitor, CID 1067700 (Agola et al., 2012) was from EMD Millipore. The NAADP antagonist trans-Ned-19 was synthesised as described previously (Naylor et al., 2009). Ned-K is an analogue of Ned-19 in which the fluoride has been replaced with a cyano group. Its synthesis will be described elsewhere. Both Ned-19 and Ned-K were kind gifts from A. Ganesan (School of Pharmacy, University of East Anglia, UK), Raj Gossain (School of Chemistry, University of Southampton, UK) and Sean M. Davidson (Hatter Institute, UCL, UK). The PIKfyve inhibitor, YM-201636 was from Cambridge Bioscience. BAPTA-AM and EGTA-AM were from Sigma.

### siRNA

Fibroblasts were transfected with siRNAs using Lipofectamine® RNAiMAX for 24 h, re-transfected for an additional 24 h and cultured for a final 24 h in the absence of siRNA prior to experimentation. A control siRNA duplex (Allstars Negative Control siRNA) and duplexes targeting human TPC1 (5′-CGAGCTGTATTTCATCATGAA-3′) (Brailoiu et al., 2009) and TPC2 (5′-CAGGTGGGACCTCTGCATTGA-3′) were purchased from Qiagen.

### Lysotracker labelling

Fibroblasts were washed three times in HEPES-buffered saline (HBS) comprising (in mM) 1.25 KH_2_PO_4_, 2 CaCl_2_, 2 MgSO_4_, 3 KCl, 156 NaCl, 10 glucose and 10 HEPES (pH 7.4; all from Sigma) and were then incubated with 100 nM Lysotracker® red (Invitrogen) for 20 min. Cells were washed again three times in HBS, and mounted in an imaging chamber (Biosciences Tools) prior to confocal microscopy.

### Immunocytochemistry

Fibroblasts were fixed for 10 min with 4% (w/v) paraformaldehyde, washed three times in phosphate-buffered saline (PBS) and then permeabilised for 10 min with 40 µM β-escin. Cells were washed again (three times in PBS), and blocked for 1 h with PBS supplemented with 1% (w/v) BSA and 10% (v/v) FBS. Fibroblasts were sequentially incubated for 1 h at 37°C with a primary anti-LAMP1 antibody (mouse, Developmental Studies Hybridoma Bank H4A3 clone supernatant; 1:10 dilution) and a secondary antibody conjugated to Alexa Fluor 647 (mouse, Invitrogen, 1:100 dilution) in blocking solution. Nuclei were labelled with 1 µg/ml DAPI (5 min). Cells were washed three times in PBS containing 0.1% (v/v) Tween® 20 in between incubations and mounted onto microscope slides with DABCO.

### Microscopy

Confocal images were captured using an LSM510 confocal scanner (Zeiss) attached to a Zeiss Axiovert 200M inverted microscope fitted with a 63× Plan Apochromat water-immersion objective. DAPI, Lysotracker® Red and Alexa Fluor 647 were excited at 364 nm, 543 nm and 633 nm, and emitted fluorescence captured using 385 nm long pass, 585–615 nm band-pass or 655–719 nm band-pass filters, respectively. Images for control and LRRK2-PD cells together with the various treatments were captured under identical acquisition settings in order to allow comparison of fluorescent intensity. Electron microscopy was performed as described previously (Tomas et al., 2004) using a JEOL 1010 transmission electron microscope.

### Quantitative PCR

Total RNA was isolated using TRIzol® (Invitrogen) according to the manufacturer's procedures. cDNA was synthesised using SuperScript® III reverse transcriptase (Invitrogen). Samples were denatured for 2 min at 94°C followed by 40 cycles of denaturation (15 s, 94°C), annealing (30 s, 60°C) and extension (30 s, 72°C) using SYBR® Green PCR mix (Invitrogen) and oligonucleotide primers designed for human TPC1 and TPC2 as previously described (Brailoiu et al., 2009). Expression levels were normalised to the expression of GAPDH following parallel amplification.

### Western blotting

Fibroblasts were harvested by scraping and lysed in Ripa buffer containing 150 mM NaCl, 50 mM Tris-HCl (pH 7.4), 0.5% sodium deoxycholic acid, 0.1% sodium dodecyl sulphate and 1% Triton X-100 in the presence of EDTA-free protease inhibitor (Roche) and Halt™ phosphatase inhibitor cocktail (Thermo Scientific) for 30 min on ice. Samples were centrifuged at 15,000 ***g*** at 4°C for 15 min and the resulting supernatants stored at −20°C until required. Samples (10–30 µg) were reduced with dithiothreitol (100 mM), separated on NuPAGE® 4–12% Bis-Tris gels (Invitrogen) and transferred onto PVDF filters (Biorad) according to standard procedures. The filters were then blocked with 5% (w/v) dried skimmed milk in Tris-buffered saline (25 mM Tris-HCl, 137 mM NaCl and 2.7 mM KCl, pH 7.4) containing 0.1% (v/v) Tween® 20 (TBS-T) for either 1 h at room temperature or overnight at 4°C. Blots were sequentially incubated with primary and secondary antibodies in TBS-T supplemented with 2.5% (w/v) dried skimmed milk. After each step, the filters were washed with TBS-T (3×30 min). The resulting blots were developed using the ECL™ Prime Western Blot Detection System (GE Healthcare) according to the manufacturer's instructions. The primary antibodies used were anti-LAMP1 (mouse, Santa Cruz Biotechnology; 1:500, overnight 4°C), anti-TPC1 (rabbit, Abcam, 1:200, 1 h room temperature), anti-VPS35 (rabbit, Abcam, 1:1000, overnight 4°C) and anti-actin (goat, Invitrogen, 1:500, 1 h room temperature) antibodies. The secondary antibodies used were anti-mouse-IgG (Santa Cruz Biotechnology), anti-rabbit-IgG (Bio-Rad) or anti-goat-IgG (Santa Cruz Biotechnology) conjugated to horseradish peroxidase (1:2000, 1 h room temperature).

### Ca^2+^ imaging and microinjection

Cytosolic Ca^2+^ concentration measurements using Fura-2 and microinjection were performed as described previously (Deliu et al., 2012).

### Data analysis

Images were analysed using ImageJ software. For Lysotracker® red and LAMP1 intensity measurements, background was subtracted from the images and mean grey intensity per cell measured within user defined regions-of-interest (comprising the whole lysosome population). Statistical analyses were performed using IBM SPSS statistics 22 software. Independent Student's *t*-tests or one-way ANOVA followed by Games–Howell post hoc tests were applied to calculate statistical significance. Values are presented as mean±s.e.m. For ANOVA analysis, threshold of significance was maintained at *P*<0.016 to correct for multiple testing error.

## Supplementary Material

Supplementary Material
