# Supplementary material for: Dysregulation of lysosomal morphology by pathogenic LRRK2 is corrected by TPC2 inhibition
Source: J Cell Sci. 2015 Jan 15;128(2):232–8. doi: 10.1242/jcs.164152 (PMC4294771; doi:10.1242/jcs.164152)
Supplement: Supplementary Material [file supp_128_2_232__index.html]

Dysregulation of lysosomal morphology by pathogenic LRRK2 is corrected by TPC2 inhibition — Supplementary Material 

# Dysregulation of lysosomal morphology by pathogenic LRRK2 is corrected by TPC2 inhibition

## JCS164152 Supplementary Material

**Files in this Data Supplement:**

- **Supplementary Material**
