# Supplementary material for: Dysregulation of lysosomal morphology by pathogenic LRRK2 is corrected by TPC2 inhibition
Source: J Cell Sci. 2015 Jan 15;128(2):232–8. doi: 10.1242/jcs.164152 (PMC4294771; doi:10.1242/jcs.164152)
Supplement: Supplementary Material [file supp_128.2.232_JCS164152.pdf]

Fig. S1. Pathogenic LRRK2 disrupts lysosomal morphology in multiple patients. A-C, LAMP1 staining in fibroblasts derived from healthy controls (left) and age-matched PD patients harbouring the LRRK2 G2019S mutation (right). Scale bars, 5 µm. **D-E**, Summary data for each control/patient pair quantifying LAMP1 intensity (**D**) or the proportion of cells displaying perinuclear lysosome clustering (**E**). Data are from 93-555 healthy control and 102-582 LRRK2-PD cells. Right panels show aggregate data for all four experimental pairs used in this study incorporating data from Fig. 1. **$p<0.01$; ***$p<0.001$

Fig. S2. Quantification of endogenous LAMP1, VPS35 and TPC levels in human fibroblasts. A, Western blot analysis using antibodies to LAMP1 (left) or VPS35 (right) and fibroblast homogenates prepared from a healthy control or LRRK2-PD patient (10 µg, LAMP1; 30 µg VPS35). Blots were stripped and re-probed using an antibody to actin (bottom panels). Migration of molecular mass markers (in kDa) is shown on the left. Results of densitometric analysis quantifying the fold-change in protein levels in PD patients versus healthy control (3 pairs, 2-5 determinations per individual) are listed at the bottom. **B**, Western blot analysis using an antibody to TPC1 and fibroblast homogenates prepared from a LRRK2-PD patient (17 µg) that were treated with a scrambled siRNA or siRNA to TPC1 or TPC2. Results of densitometeric analysis quantifying the fold-change in TPC1 levels (bracketed area) in TPC siRNA-treated cells relative to scrambled siRNA (3 independent knock downs from 2 patients).

Fig. S3. Lysosomal defects are reversed by acute inhibition of LRRK2 kinase, Rab7 and NAADP. A-H. LAMP1 staining in fibroblasts from a healthy control (**A**) and a PD patient (**B-H**) treated for the indicated time with LRRK2-In1 (100 nM, **C-D**), the Rab7 GTPase

inhibitor, CID 1067700 (1 µM, **E**), Ned-19 (100 µM, **F-G**) and Ned-K (100 µM**, H**)**.** Scale bars, 5 µm. **I,** Pooled data (n=50-78 cells). *** p<0.001.

Fig. S4. Pathogenic LRRK2 disrupts lysosomal ultrastructure. Electron micrograph of a LRRK2-PD fibroblast. Example of an hour-glass structure (*). Scale bar, 500 nm.

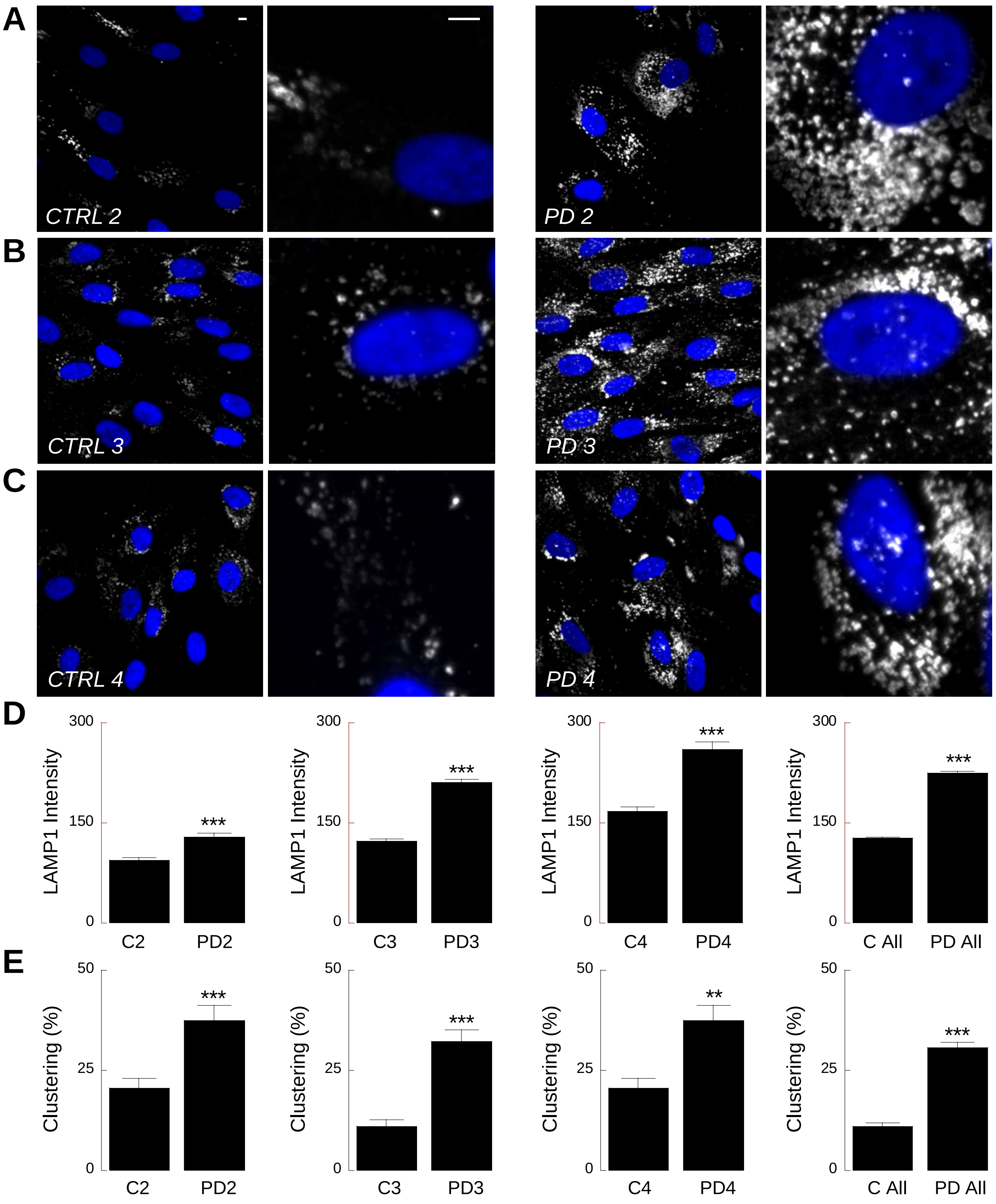
A
CTRL 2
PD 2
B
CTRL 3
PD 3
C
CTRL 4
PD 4
D
LAMP1 Intensity
300
150
0
C2
PD2
***
C3
PD3
***
C4
PD4
***
C All
PD All
***
E
Clustering (%)
50
25
0
C2
PD2
***
C3
PD3
***
C4
PD4
**
C All
PD All
***

Figure S1

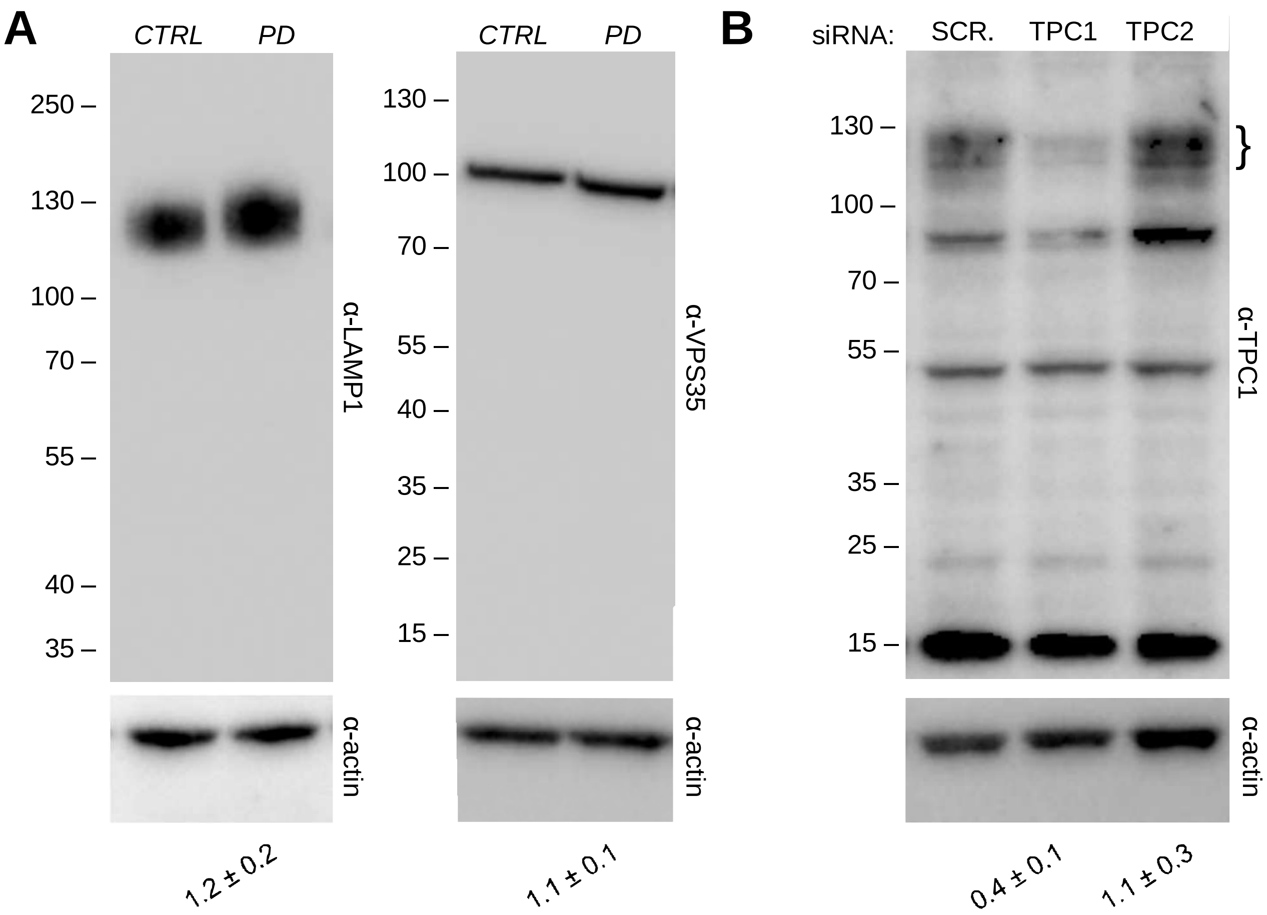
A
CTRL
PD
250 –
130 –
100 –
70 –
55 –
40 –
35 –
α-LAMP1
α-actin
1.2 ± 0.2
CTRL
PD
130 –
100 –
70 –
55 –
40 –
35 –
25 –
15 –
α-VPS35
α-actin
1.1 ± 0.1
B
siRNA:
SCR.
TPC1
TPC2
130 –
100 –
70 –
55 –
35 –
25 –
15 –
α-TPC1
α-actin
0.4 ± 0.1
1.1 ± 0.3

Figure S2

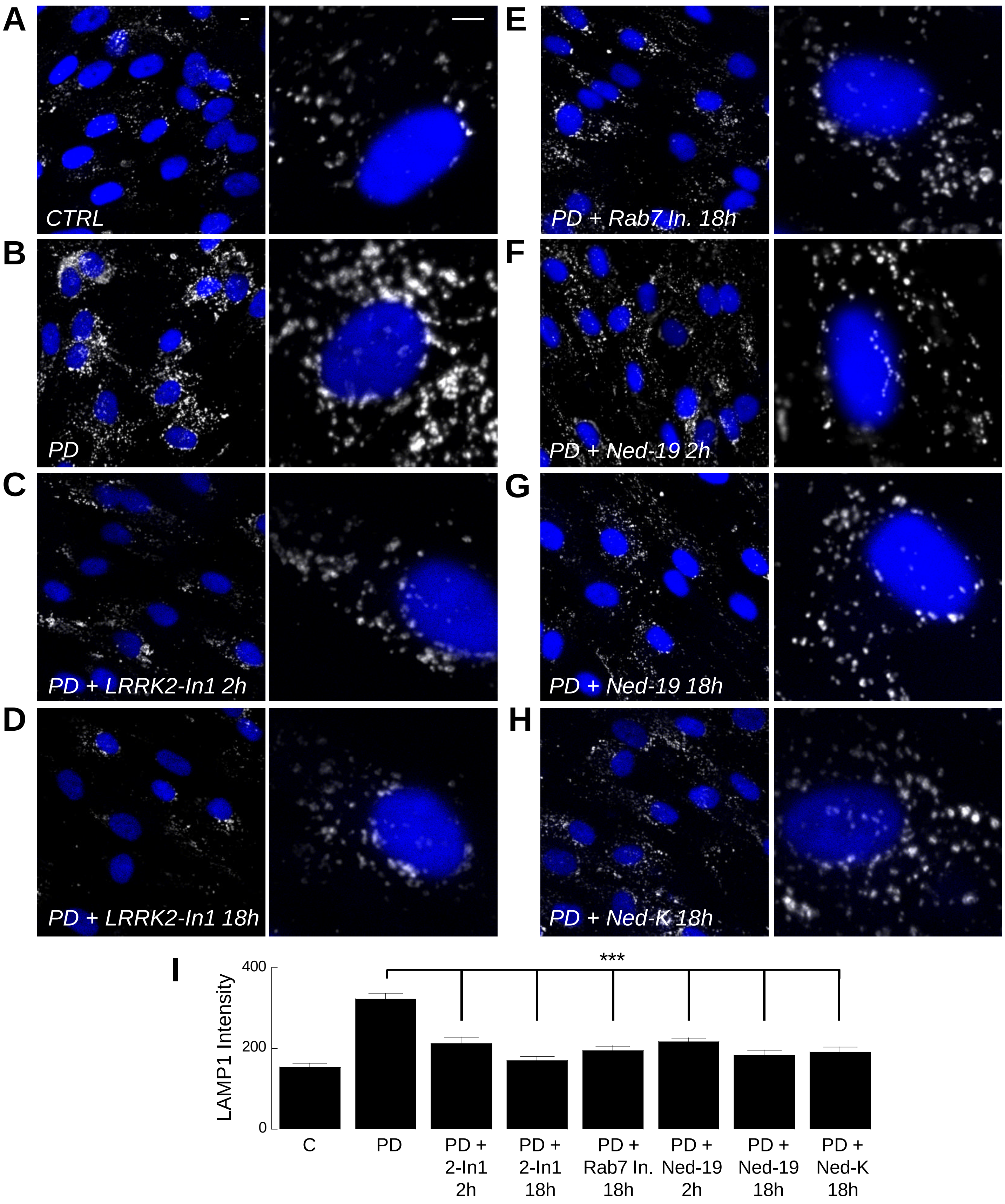
A
CTRL
B
PD
C
PD + LRRK2-In1 2h
D
PD + LRRK2-In1 18h
E
PD + Rab7 In. 18h
F
PD + Ned-19 2h
G
PD + Ned-19 18h
H
PD + Ned-K 18h
I
LAMP1 Intensity
400
200
0
***
C
PD
PD + 2-In1 2h
PD + 2-In1 18h
PD + Rab7 In. 18h
PD + Ned-19 2h
PD + Ned-19 18h
PD + Ned-K 18h

Figure S3

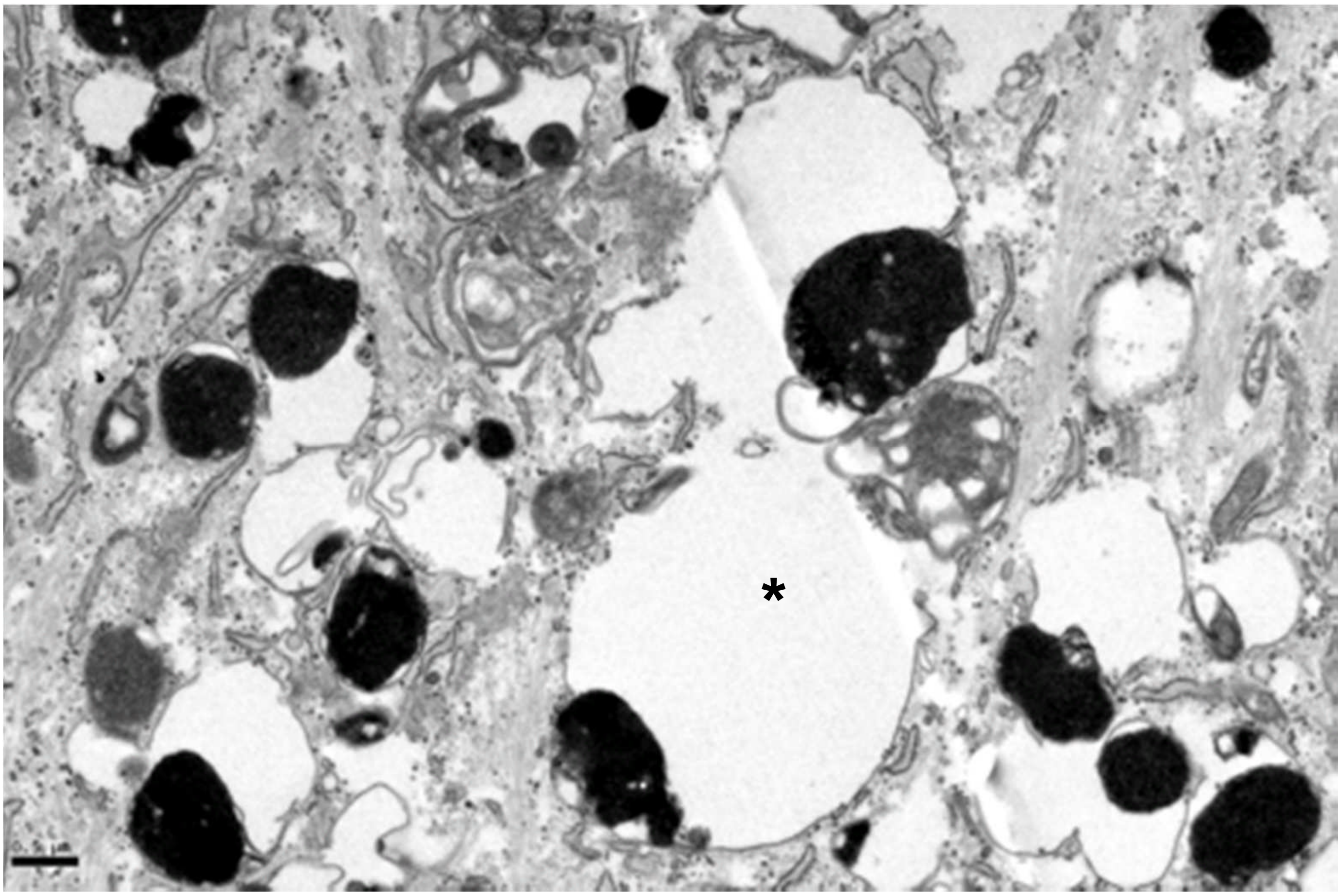

Figure S4
